# Population pharmacokinetic analysis, renal safety, and dosing optimization of polymyxin B in lung transplant recipients with pneumonia: A prospective study

**DOI:** 10.3389/fphar.2022.1019411

**Published:** 2022-10-13

**Authors:** Xiao-Jun Cai, Yan Chen, Xiao-Shan Zhang, Yu-Zhen Wang, Wen-Bo Zhou, Chun-Hong Zhang, Bo Wu, Hui-Zhu Song, Hang Yang, Xu-Ben Yu

**Affiliations:** ^1^ Department of Pharmacy, The Affiliated Wuxi People’s Hospital of Nanjing Medical University, Wuxi, China; ^2^ Division of Pharmacy, Wuxi Higher Health Vocational Technology School, Wuxi, China; ^3^ Department of Pharmacy, The First Affiliated Hospital of Wenzhou Medical University, Wenzhou, China; ^4^ School of Pharmacy, Wenzhou Medical University, Wenzhou, China; ^5^ Lung Transplant Center, The Affiliated Wuxi People’s Hospital of Nanjing Medical University, Wuxi, China; ^6^ School of Pharmacy, Chonnam National University, Gwangju, South Korea

**Keywords:** polymyxin B, population pharmacokinetics, lung transplantation, dosing optimization, renal function

## Abstract

**Objectives:** This study aims to characterize the population pharmacokinetics of polymyxin B in lung transplant recipients and optimize its dosage regimens.

**Patients and methods:** This prospective study involved carbapenem-resistant organisms-infected patients treated with polymyxin B. The population pharmacokinetic model was developed using the NONMEM program. The clinical outcomes including clinical treatment efficacy, microbiological efficacy, nephrotoxicity, and hyperpigmentation were assessed. Monte Carlo simulation was performed to calculate the probability of target attainment in patients with normal or decreased renal function.

**Results:** A total of 34 hospitalized adult patients were included. 29 (85.29%) patients were considered of clinical cure or improvement; 14 (41.18%) patients had successful bacteria elimination at the end of the treatment. Meanwhile, 5 (14.71%) patients developed polymyxin B-induced nephrotoxicity; 19 (55.88%) patients developed skin hyperpigmentation. A total of 164 concentrations with a range of 0.56–11.66 mg/L were obtained for pharmacokinetic modeling. The pharmacokinetic characteristic of polymyxin B was well described by a 1-compartment model with linear elimination, and only creatinine clearance was identified as a covariate on the clearance of polymyxin B. Monte Carlo simulations indicated an adjusted dosage regimen might be needed in patients with renal insufficiency and the currently recommended dose regimens by the label sheet of polymyxin B may likely generate a subtherapeutic exposure for MIC = 2 mg/L.

**Conclusion:** Renal function has a significant effect on the clearance of polymyxin B in lung transplant recipients, and an adjustment of dosage was needed in patients with renal impairments.

## 1 Introduction

Polymyxins (colistin and polymyxin B) are being used against gram-negative bacteria, such as *Escherichia coli, Klebsiella pneumoniae, Acinetobacter baumannii*, and *Pseudomonas aeruginosa* since the 1960s (Evans et al., 1999; Mohapatra et al., 2021). However, its side effects, such as nephrotoxicity and hyperpigmentation, limited its clinical use. Recently, polymyxins are currently used as one of the last-line options for the treatment of carbapenem-resistant organisms (CRO) infections (Tsuji et al., 2019).

Due to the frequent use of antimicrobials, prolonged hospitalization, and the immune-suppression state, multidrug-resistant (MDR) gram-negative bacterial infection is a serious threat to the recipients with solid organ transplantation and hematopoietic stem cell transplantation (Dobbin et al., 2004; Linares et al., 2007; Moreno et al., 2007), which generates an increasing need for polymyxins use. Infections are the most frequent complication in lung transplant recipients (up to 42% within 3 months), with the respiratory tract being the most common site of infection in the early posttransplant period (Burguete et al., 2013; Alsaeed and Husain, 2019). Moreover, MDR organisms have recently been considered as an increased risk of early transplant morbidity and mortality (Dominguez and Blodget, 2020).

Polymyxin B can be administered intravenously, intrathecally, or by aerosol inhalation (Tsuji et al., 2019), with a half-life of about 9–11.5 h after being administered intravenously, and a steady-state volume of distribution of about 12.7–34.3 L (Chen et al., 2022). The urinary recovery of polymyxin B is low and non-renal clearance is considered the major route of the clearance of polymyxin B (Tsuji et al., 2019), but it undergoes extensive reabsorption by renal tubular cells (Nang et al., 2021). The PKPD result from mouse infection studies indicates that parenteral polymyxin B cannot be efficacious against pulmonary infections due to poor penetration in the lungs (Nang et al., 2021). Therefore, the combination treatment of parenteral administration and aerosol inhalation of polymyxin is recommended (Tsuji et al., 2019).

It is generally acknowledged that the pharmacokinetics of patients with solid organ transplantations are different from those of normal patients (Han et al., 2010; Lin et al., 2018; Li et al., 2021), which often manifest extreme pathophysiological changes that may affect the PK of antibiotics, such as changes in the volume of distribution, protein binding, and extracorporeal clearance. Recently, Li et al. (2021) investigated the population pharmacokinetics of polymyxin B in renal transplant recipients, who found that the clearance of polymyxin B was lower than that in normal patients, and a reduced dosage regimen was recommended in those patients with renal impairment. However, no pharmacokinetic and pharmacodynamics (PK/PD) data on polymyxin B has been conducted yet for the recipients with lung transplantation, and the optimal dosing remains unclear. To the best of our knowledge, data on the clinical efficacy and adverse effects of polymyxin B in lung transplant recipients also have not been reported.

In the current study, we first aimed to develop a population PK model to describe polymyxin B pharmacokinetics in lung transplant recipients and identify the variability. In addition, the clinical efficacy and adverse events of polymyxin B during the treatment were evaluated. Finally, the probability of PK/PD target attainment of polymyxin B with the dose regimens recommended by the label sheet was assessed to facilitate its dose individualization.

## 2 Materials and methods

### 2.1 Patients and ethics

This prospective study was approved by the Ethical Committees of the affiliated Wuxi People’s Hospital of Nanjing Medical University, China (KS202002), in accordance with the Declaration of Helsinki. The informed consent was obtained from each patient or his/her legal representatives. Adult lung transplant recipients receiving polymyxin B sulfate against confirmed MDR Gram-negative bacterial infections at the affiliated Wuxi People’s Hospital of Nanjing Medical University, from January 2020 to December 2021, were included in this study. The inclusion criteria were as follows: 1) ≥18 years old; 2) diagnosis of pneumonia; 3) receiving polymyxin B therapy for at least 3 days; 4) having at least one plasma concentration of polymyxin B. The exclusion criteria were as follows: 1) allergic or intolerant to polymyxin B; 2) died within 24 h after being treated with polymyxin B.

### 2.2 Clinical data collection

The demographic characteristics, laboratory data, pathogenic bacteria, medication information, and adverse events of polymyxin B sulfate were acquired from the patients’ medical records. Clinical success was defined as improvement of clinical symptoms and parameters including body temperature, biochemistry indicators of infection (white cell count ≤ 10^9^, C-reactive protein ≤10 mg/L, procalcitonin <0.05 ng/ml, and erythrocyte sedimentation rate <15 mm/h), and clinician-documented improvement at the end of treatment (Yu et al., 2022). RIFLE (Risk, Injury, Failure, Loss of function, and End-stage kidney disease) criteria were used to assess the acute kidney injury (AKI) caused by polymyxin B (Han et al., 2022). The definition of nephrotoxicity caused by polymyxin B was further confirmed using the Naranjo criteria. Hyperpigmentation is defined as the darkening of the skin’s natural color, usually due to an increase in melanin deposition in the epidermis or dermis (Lu and Hou, 2020).

### 2.3 Polymyxin B administration and sample collection

The decision to administer polymyxin B (sulfate, Polymyxin B for Injection, Shanghai Number one Biochemical and Pharmaceutical, Shanghai, China) and its medication, including the amount, dosing interval, administration route, and treatment duration, were decided by the attending physician. Generally, the loading dose of 2.0–2.5 mg/kg, and the maintenance dose of 1.25–1.5 mg/kg given every 12 h infused over 1 h was recommended according to the guideline for optimizing polymyxins (Tsuji et al., 2019). In addition, inhalation of polymyxin B sulfate (25 mg q12h) was combinedly used.

All the blood samples were collected at least 48 h after initiating polymyxin B (Tsuji et al., 2019). In each patient, four blood samples were collected: 0.5 h before starting the infusion, 1, 2, and 6 h after completing the infusion, respectively. After sampling, blood samples were immediately centrifuged at 15,000 rpm for 5 min and stored at −80°C. The concentrations of polymyxin B in the blood samples were determined by a validated high-performance liquid chromatography-tandem mass spectrometry (LC-MS/MS) assay (Thomas et al., 2012). The calibration range of polymyxin B by the method was 0.1–30 μg/ml. The method validations including selectivity, recovery, precision, accuracy, matrix effect, calibration curve, and stability met the requirement of FDA principles.

### 2.4 Population pharmacokinetic modeling of polymyxin B

Nonlinear mixed-effects modeling (NONMEM) program (version 7.4, Icon Development Solutions, Ellicott City, MD, United States) and Pirana (version 2.9.7) was used to perform PK analysis of polymyxin B. R (Version 3.6.1) was used to analyze the NONMEM output. The first-order conditional estimation with the interaction between inter-patient variability and residual variability was used for model development.

#### 2.4.1 Base model

One- or two-compartment structural model with first-order elimination were explored for the concentration-time profiles. Visual inspection of diagnostic plots and goodness-of-fit criteria, condition number, and improvement of the objective function value (OFV) were performed to evaluate the base model. The pharmacokinetic parameters used for this study were clearance (CL) and volume of distribution (V). Between-subject variability (BSV) was assessed using the exponential model (Eq. 1).

Pi=P×EXP(ETA( ηi)) (1)

Pi was the individual patient PK parameter; P was the typical value; ηi was the interpatient random variation.

Residual variability was assessed by additive (Eq. 2), proportional (Eq. 3), or combined (additive plus proportional) (Eq. 4) error models, respectively. The optimal one was chosen based on the decrease in OFV.

Y=F+EPS (1) (2)

Y=F+F×EPS (1) (3)

Y=F+F×EPS (1)+EPS(2) (4)

Y was the individual observed concentration; F was the individual predictive concentration; EPS was the residual random variation.

#### 2.4.2 Covariate model

Relationships between the potential covariates and individual PK parameters were examined visually. The potential covariates include age, gender, body weight, height, hemoglobin (Hb), white blood cell count (WBC), percentage of neutrophils (N%), platelet count (PLT), aminotransferase (ALT), aspartate aminotransferase (AST), total bilirubin (TBIL), alanine serum albumin (ALB), serum total protein (TP), C-response protein (CRP), procalcitonin (PCT), blood urea nitrogen (BUN), serum creatinine (SCr), creatinine clearance (CrCL), concomitant treatment with furosemide or albumin during polymyxin B treatment. Before developing the covariate model, Pearson’s test was performed to identify the correlation between the candidate covariates, and the one with greater OFV decreasing was chosen for analysis. The stepwise forward selection was performed to include the potential covariates until no further decrease in OFV ( ∆OFV ) was observed. Specifically, each covariate was incorporated in the base model, and the one with a decrease in ∆OFV greater than 3.84 (*p* < 0.05) was considered significant. The included covariates were further assessed using backward deletion, the incorporated covariates were retained when the increase in OFV was greater than 6.63 (*p* < 0.01). Additional criteria, such as biological plausibility, increase in PK parameter estimate precision, and decrease in unexplained BSV, were considered for retaining the covariate in the final model.

#### 2.4.3 Model evaluation

Goodness-of-fit plots, nonparametric bootstrap, prediction- and variability-corrected visual predictive check (pvcVPC), and normalized prediction distribution error (NPDE) methods were employed to evaluate the final model.

Nonparametric bootstrap was performed to assess the stability and robustness of the final model. Specifically, 1,000 replicate datasets were generated from random sampling with replacement using the individual as the sampling unit. The PK parameters were then calculated for each dataset, and the median and 95% confidence intervals of these parameters were compared with the final parameter estimates.

For the pvcVPCs test, datasets were simulated 1,000 times using the PsN^®^ to produce the concentration data. The statistics of the observed and simulated concentration-time profiles were compared using pvcVPC, and the 5th, 50th, 95th percentiles of the simulated concentrations were plotted overlaid with the observed concentrations.

For the NPDE test, 1,000 simulations were generated for each observation in the raw dataset. The results were summarized graphically and statistically with the NPDE package using the R^®^. Plot of NPDE symmetrically distribution, histogram plot of NPDE distribution, plot of NPDE versus predictive concentrations, and plot of NPDE versus time, were performed to assess the final model. The Fisher’s variance test was performed to assess the statistical difference between the variance of NPDE from 1. The *t*-test was performed to assess the statistical difference between the mean value of NPDEs from 0. The Shapiro-Wilk test was performed to assess the symmetry distribution of NPDE.

### 2.5 Monte Carlo simulations

Monte Carlo simulations were performed based on the final model to identify the pragmatic dose regimen of polymyxin B in the lung transplant recipients. The ratio of the area of unbound concentration-time curve to the MIC (fAUC/MIC) is the best PK/PD index predicting bacterial killing for polymyxins (Dudhani et al., 2010a; Dudhani et al., 2010b; Cheah et al., 2015). The fAUC/MIC value ≥20 at various MICs (0.5–2 mg/L) was used as the target (Sandri et al., 2013), where f (assumed to be 0.42) is the unbound fraction of polymyxin B (Sandri et al., 2013). The probability of target attainment (PTA) was calculated for each dose regimen (40 mg q12h, 50 mg q12h, 75 mg q12h, 100 mg q12h, with a loading dose of 2 × maintenance dose). The cut-off value for the PTA was set at 80% (Wu et al., 2022). The AUC_0-24_ was calculated by the linear-log trapezoidal rule using the concentrations at continuous time (10 min-interval) predicted *via* Bayesian estimation. Specifically, the linear trapezoidal approach was used during the ascending phase and the log-linear method was used during the descending phase (Wu et al., 2022).

AUC_ss,24h_ were calculated for each simulated dosing regimen in various renal function patients. AUC_ss,24h_ below 100 mg h/L was taken as the safety target according to the current guideline for optimal use of polymyxins (Tsuji et al., 2019).

## 3 Results

### 3.1 Characteristics of patients

A total of 34 adult patients were included according to the inclusion and exclusion criteria. The clinical characteristic data, including the demographic, laboratory data, and pathogenic bacterial information are summarized in Table 1. Most patients were male (25/34) and elderly people with a mean ± S.D. age of 52.15 ± 10.00 years. In addition, the highest proportion of the isolated CRO was *A. baumannii* (47.5%), followed by *P. aeruginosa* (30%), *K. pneumoniae* (17.5%), and *Enterobacter cloacae* (5%).

**TABLE 1:** Clinical characteristics of recipients with lung transplantation.

Characteristic	Value^a^
Age (years)	56 ± 12.76
Gender (male/female)	25/9
Body weight (kg)	52.15 ± 10.00
Body height (cm)	166.93 ± 7.20
Laboratory data	
White cell count (×10^9^/L)	13.62 ± 6.48
Hemoglobin (g/L)	97.86 ± 16.47
Platelet (×10^9^/L)	180.31 ± 96.95
Total bilirubin (μmol/L)	16.8 [10.65, 23.5]
Alanine transaminase (U/L)	19.99 ± 19.03
Aspartic transaminase (U/L)	23.66 ± 13.00
Serum albumin (g/L)	33.15 ± 5.16
Serum creatinine (μmol/L)	72.40 ± 33.45
Creatinine clearance (ml/min)	80.81 ± 29.97
Pathogenic bacteria	
*A. baumannii*	19 (47.5%)
*K. pneumoniae*	7 (17.5%)
*P. aeruginosa*	12 (30%)
*E. cloacae*	2 (5%)
Treatment duration (days)	17.94 ± 7.13
Daily dose of intravenous adiministration (IU)	100 [100, 150]
Daily dose of aerosol inhalation (IU)	50 [50, 50]

^a^ Values are No. (%) or median [min, max] or mean ± SD.

### 3.2 Medications and outcomes

All patients had been treated with polymyxin B sulfate intravenously and were combined with inhaling polymyxin B. The treatment duration was 17.94 ± 7.13 days. 29 (85.29%) patients were considered of clinical cure or improved, and 41.18% of patients achieved bacteria elimination at the end of treatment. A total of 5 (14.71%) patients developed polymyxin B-related nephrotoxicity, while 19 (55.88%) patients developed skin hyperpigmentation (Table 2).

**TABLE 2:** Total outcomes of recipients with lung transplantation.

Outcome	Value^a^
AUC (mg·h/L)	69.66 ± 35.92
Clinical anti-infective outcome	
Cured	12 (35.29%)
Improved	17 (50%)
Failed	5 (14.71%)
Bacteria elimination (yes)	14 (41.18%)
Nephrotoxicity (yes)	5 (14.71%)
Skin hyperpigmentation	19 (55.88%)

^a^ Values are No. (%) or mean ± SD.

### 3.3 Population pharmacokinetic analysis

**FIGURE 1:**
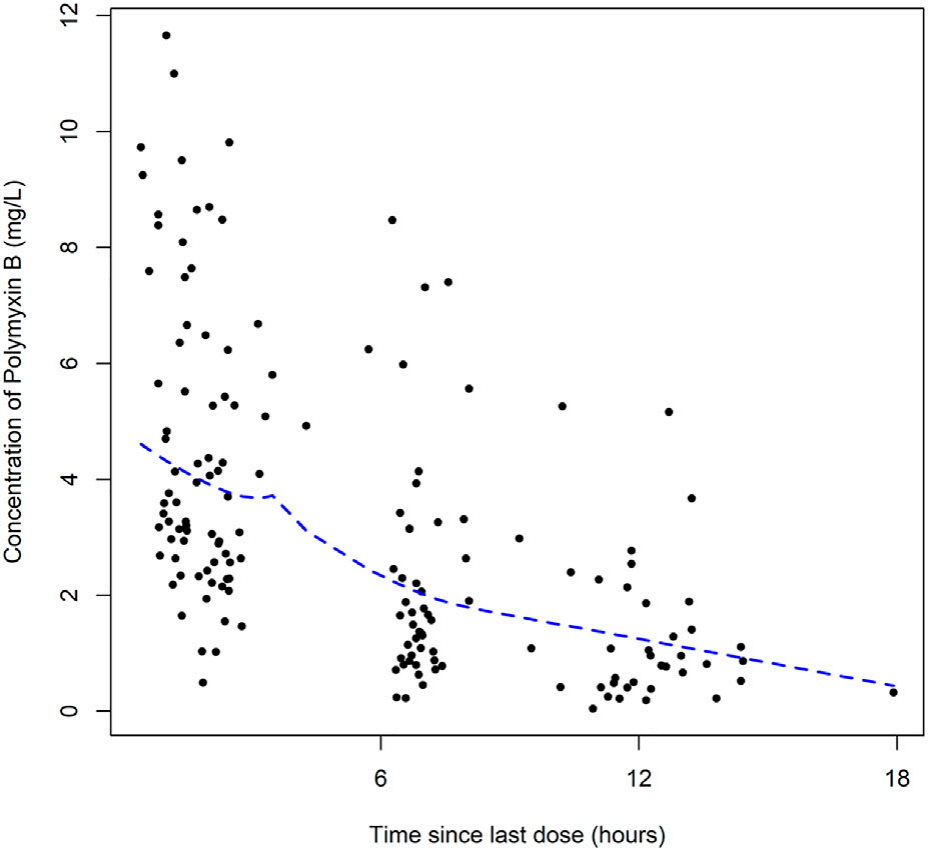
Dose-normalized serum concentration-time profiles of polymyxin B.

A total of 164 concentrations with a range of 0.56–11.66 mg/L were obtained for PK modeling of polymyxin B in lung transplant recipients. There is no concentration data obtained from patients during extracorporeal membrane oxygenation therapy or renal replacement therapy. The plots of polymyxin B concentration versus the time after the last dose was shown in Figure 1.

For the base model, the one-compartment model with first-order elimination showed a better fit of the observed concentration-time data compared to the 2-compartment model. The BSV was successfully estimated for the parameter of both CL and V. Proportional error model was selected to assess the residual variability. The diagnostic plots and the parameter estimates for the base model were shown in Supplementary Figure S1 and Supplementary Table S1.

The potential covariates were tested on both CL and V. Finally, CrCL was identified as a significant covariate on the CL of polymyxin B. The final population PK model is represented as follows:

CL(L/h)=1.72+(CrCL78.49)^0.681 (5)

V(L)=14.4 (6)

Where *CL* is the clearance, *V* is the distribution volume, and *CrCL* is the estimated creatinine clearance. 1.72 is the typical value of clearance, 78.49 is the median value of *CrCL* for the included patients in this study, 0.681 is the exponential value for *CrCL* as a covariate on *CL*, and 14.4 is the typical value of the volume of distribution. The parameter estimates of the final model were shown in Table 3.

**TABLE 3:** Population pharmacokinetic parameter estimates from the final model and bootstrap results.

Parameter	Final model		Bootstrap of final model			Bias (%)
	Estimate	RSE (%)	2.5th percentile	Median estimate	97.5th percentile	
θ_CL_[L/h]	1.72	8	1.44	1.72	1.99	−0.24
θ_V_[L]	14.4	11	11.4	14.3	17.4	−0.24
CrCL on CL (θ_1_)	0.681	20	0.360	0.686	1.002	0.70
Between-subject variability						
ω_CL_ [%]	32.6	12	23.3	31.5	39.6	−3.32
ω_V_ [%]	40.6	17	22.5	39.1	52.9	−3.59
Residual variability						
σ_pro_(%)	37.9	15	31.4	37.8	43.6	−0.2

RSE (%), relative standard error; θ_CL_, typical value of clearance; θ_V_, typical value of volume of distribution; θ_1_, exponential value for CrCL as covariate of CL; ω_CL_, square root of between-subject variability for CL; σ_pro_, proportional error. Bias (%) = (Median estimate_Bootstrap_ − Estimate_Final model_)/Estimate_Final model_ × 100.

**FIGURE 2:**
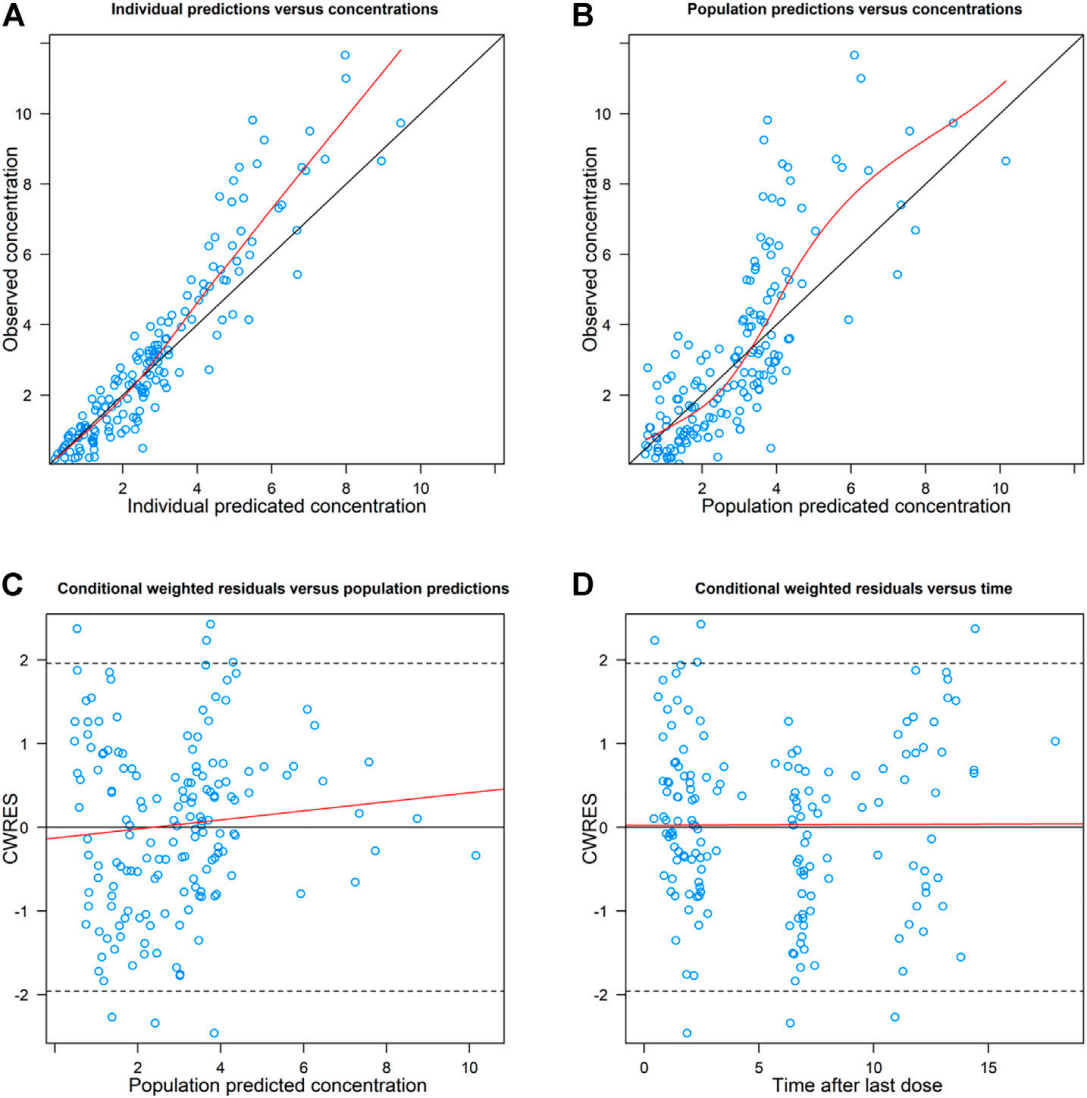
Goodness-of-fit plots of the final model. **(A)** Observed concentration (DV) versus individual predicted concentration (IPRED); **(B)** DV Versus population predicted concentration (PRED); **(C)** conditional weighted residuals (CWRES) versus PRED; and **(D)** CWRES Versus time after the last dose. The red solid lines in **(A)** and **(B)** are identity lines, and the red solid lines in **(C)** and **(D)** are zero lines.

**FIGURE 3:**
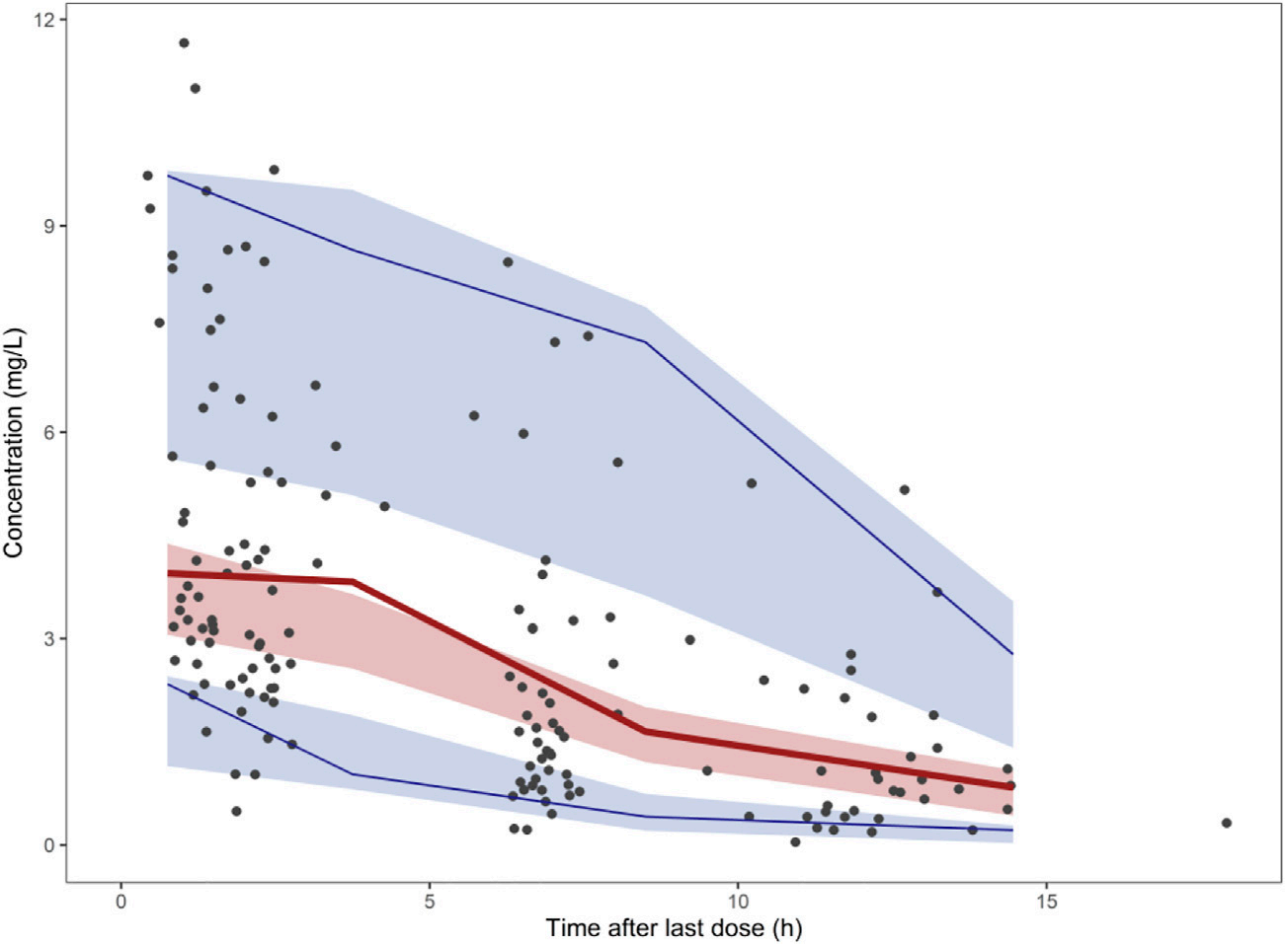
Prediction- and variability-corrected visual predictive check (pvcVPC) plot of the final model. The red solid lines represent the median observed concentration, and the semitransparent red fields represent the simulation-based 95% confidence intervals (CIs) for the median. The observed 5th and 95th percentiles are represented by red dashed lines, and the 95% CIs for the corresponding model predicted percentiles are shown as semitransparent blue fields. The observed concentrations are represented by dark dots.

The goodness-of-fit plots of the final model were shown in Figure 2. The CWRES vs. PRED of the final model showed a stochastic distribution around 0, with most residuals within −2 to 2. In addition, the bootstrap analysis showed that the parameter estimates of the final model laid within the 95% CI parameter estimates resulted from the nonparametric bootstrap analysis, and the biases were < ±10% (Table 3), indicating good stability of the final model. The results of NPDE indicated good prediction performance for the final model (Supplementary Figure S2). The *p* values of the *t*-test and Fisher’s variance test were 0.887 and 0.323, respectively, suggesting that the NPDE had a mean of 0 and a variance of 1. The *p*-value of the Shapiro-Wilks test was 0.765, suggesting a symmetrical distribution of NPDE. The pvcVPC profile of concentrations versus TAD showed a good fit between simulations and the observations (Figure 3). Overall, the final PK model provided an adequate description of the data and a good prediction of individual PK parameters of polymyxin B in lung transplant recipients.

### 3.4 Monte Carlo simulations

**FIGURE 4:**
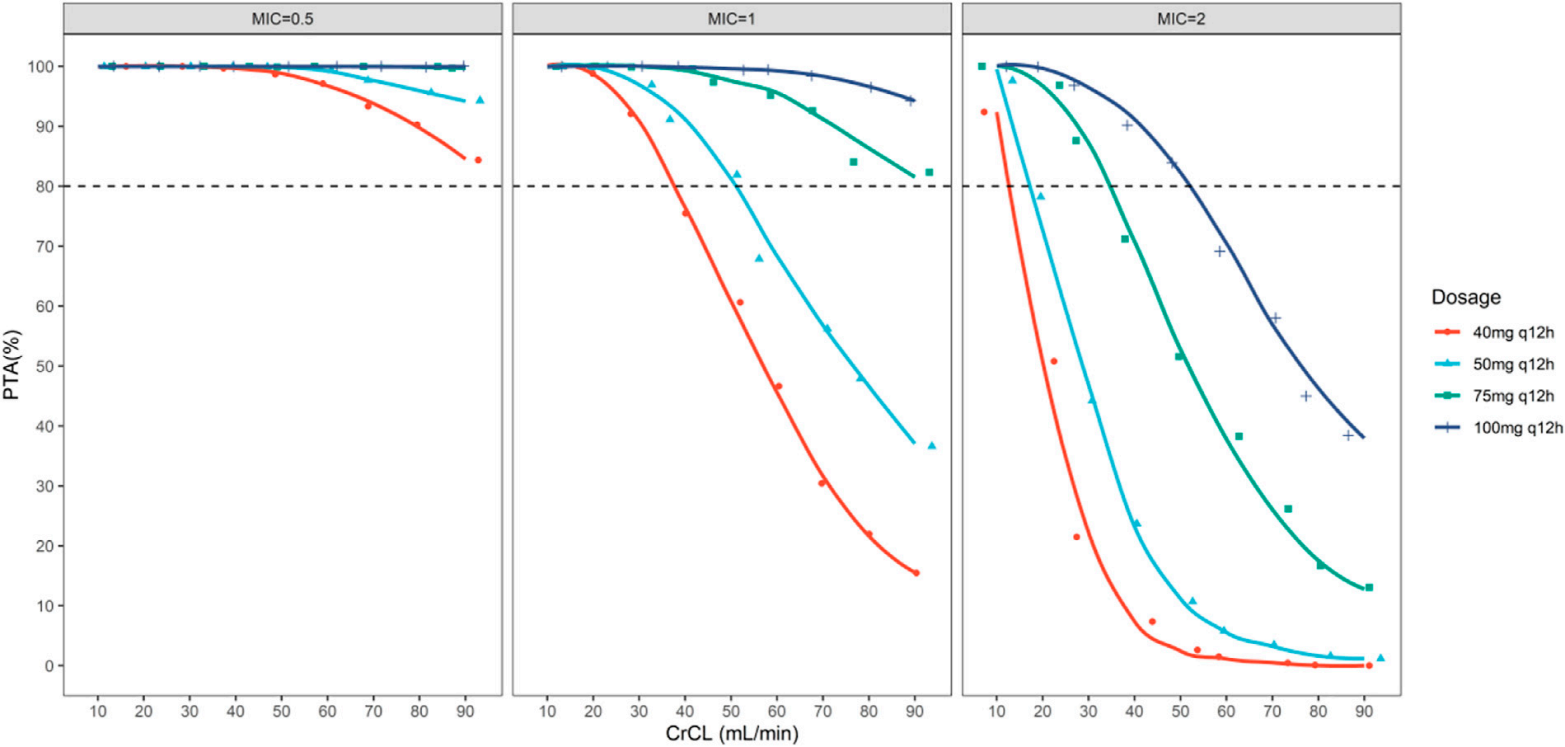
The simulated probability of target attainment (efficacy target of fAUC/MIC ≥20) of polymyxin B on 40, 50, 75, and 100 mg q12h regimens in lung transplant patients with different creatinine clearance.

The PTA for the different dosage regimens of polymyxin B within the fAUC/MIC ≥20 at day 3 was shown in Table 4 and Figure 4. For MIC = 0.5 mg/L, all of the regimens could achieve the PTA in all groups. For MIC = 1 mg/L, 40 mg 12 h was of underexposure in patients with CrCL >30 ml/min; 50 mg q12h was of underexposure in patients with CrCL >50 ml/min; 75 mg q12h and 100 mg q12h could achieve PTA in all groups. However, most of the simulated dosage regimens could not achieve the PTA for MIC at the current EUCAST breakpoint of 2 mg/L, except the regimen of 100 mg q12h in patients with CrCL <50 ml/min and the regimen of 75 mg q12h in patients with CrCL <30 ml/min.

**TABLE 4:** Probability of target attainment based on different CrCL and dosage regimens.

CrCL (ml/min)	MIC (mg/L)	Dosage^a^ of PTA (mg)			
		80 + 40	100 + 50	150 + 75	200 + 100
10	0.5	1	1	1	1
	1	1	1	1	1
	2	0.924	0.976	1	1
20	0.5	1	1	1	1
	1	0.988	0.998	1	1
	2	0.508	0.782	0.969	0.999
30	0.5	1	1	1	1
	1	0.921	0.969	0.999	1
	2	0.215	0.442	0.876	0.968
40	0.5	0.997	1	1	1
	1	0.755	0.911	0.996	1
	2	0.074	0.237	0.712	0.902
50	0.5	0.987	0.999	0.999	1
	1	0.606	0.819	0.974	0.994
	2	0.026	0.107	0.516	0.839
60	0.5	0.971	0.993	1	1
	1	0.466	0.679	0.952	0.994
	2	0.015	0.058	0.383	0.691
70	0.5	0.933	0.977	1	1
	1	0.304	0.562	0.926	0.984
	2	0.004	0.035	0.262	0.58
80	0.5	0.902	0.956	0.999	0.999
	1	0.22	0.479	0.841	0.965
	2	0.001	0.016	0.167	0.45
90	0.5	0.844	0.943	0.997	1
	1	0.155	0.366	0.823	0.943
	2	0	0.012	0.131	0.384

^a^ Loading dose + maintenance dose twice daily.

CrCL, creatinine clearance calculated using the Cockcroft–Gault equation; MIC, minimum inhibitory concentration; PTA, probability of target attainment.

Gray background, groups that did not reach the PTA target.

**FIGURE 5:**
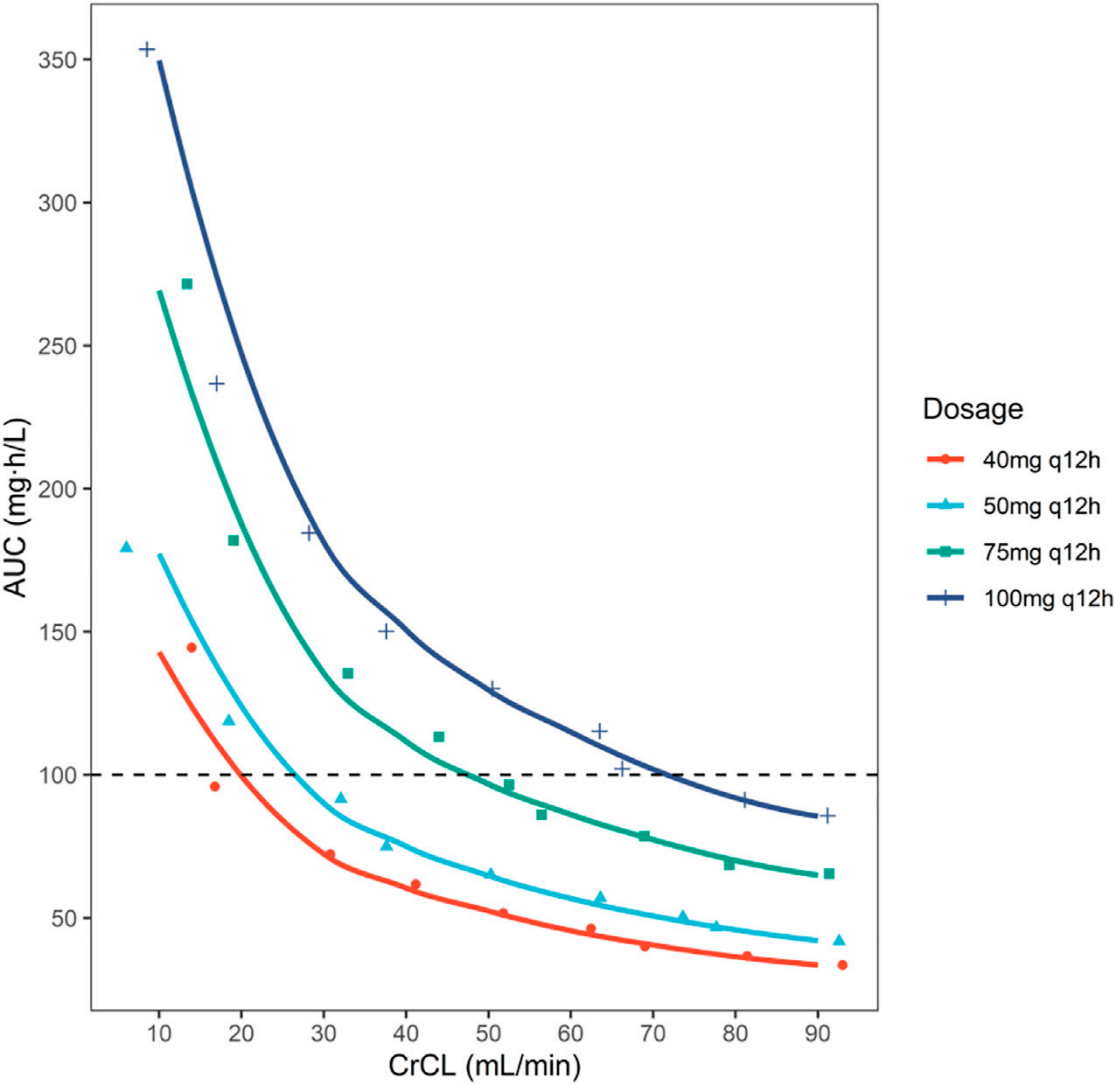
The simulated AUC_ss,24h_ of polymyxin B on 40, 50, 75, and 100 mg q12h regimens in lung transplant patients with different creatinine clearance. Safety target was set as AUC_ss,24h_ < 100 mg h/L.

The current guideline for optimal use of polymyxin B (Tsuji et al., 2019) recommended AUC_ss,24h_ < 100 mg h/L as the safety target. We calculated AUC_ss,24h_ for each simulated dosing regimen in various renal function patients. As shown in Figure 5, patients with CrCL <70 ml/min were of risking nephrotoxicity under dosage of 100 mg q12h; patients with CrCL <50 ml/min were of risking nephrotoxicity under dosage of 75 mg q12h; patients with CrCL <30 ml/min were of risking nephrotoxicity under dosage of 50 mg q12h; patients with CrCL <20 ml/min were of risking nephrotoxicity under dosage of 40 mg q12h.

In general, for patients with CrCL <20 ml/min, the current simulated dosage regimens were not recommended as the high risk of nephrotoxicity.

For patients with CrCL of 20–30 ml/min, though a decreased dosage of 40 mg q12h could reduce risking of nephrotoxicity, the patients were risking underexposure as the renal function of patients might be changing frequently. For patients with CrCL of 30–50 ml/min, 50 mg q12h was recommended, as this regimen could achieve PTA target for MICs ≤1 mg/L while within the safety target. Similarly, for patients with CrCL of 50–90 ml/min, 75 mg q12h was recommended. In addition, although the regimen of 100 mg q12h could achieve the PTA for MIC = 2 mg/L in patients with CrCL <50 ml/min; and 75 mg q12h could achieve the PTA for MIC = 2 mg/L in patients with CrCL <30 ml/min, it was of high risk of nephrotoxicity as its AUC_ss,24h_ exceeded the safety target. Thus, though a high daily dose would be possible for treating infections caused by organisms with MIC = 2 mg/L, the risk of nephrotoxicity is greatly increased.

## 4 Discussion

Polymyxin is one of the last-line options in the treatment of life-threatening infections caused by CRO (Mohapatra et al., 2021). Patients receiving lung transplantation are at high risk of CRO infections (Trachuk et al., 2020). However, studies on the pharmacokinetics, efficacy, and safety of polymyxin B in lung transplant recipients, are lacking. To the best of our knowledge, this is the first study that investigated the PK profiles of polymyxin B and optimized the dosage regimens in lung transplant recipients. Our results suggested that renal function was a significant covariate on the clearance of polymyxin B in lung transplant recipients, and dosage regimen adjustment was needed in those patients with renal impairments.

In this study, we developed a PK model to characterize the pharmacokinetics of polymyxin B for the first time in lung transplant recipients. The CL was estimated at 1.72 L/h, and the V was estimated at 14.4 L in the population. The CL of polymyxin B in this study was similar to the one we published previously in critically ill patients (Yu et al., 2021) and another study investigated in Chinese adult patients (Wang et al., 2020), whereas it was lower than the one reported in non-Chinese patients (Manchandani et al., 2018; Miglis et al., 2018) (Supplementary Table S2). In addition, CrCL was identified as the significant covariate on polymyxin B clearance in the lung transplant recipients, indicating that renal function could affect polymyxin B exposure. A potential relationship between CrCL and polymyxin B clearance has been identified in several studies (Avedissian et al., 2018; Wang et al., 2020; Yu et al., 2021). Currently, it is still a dispute that whether the renal function has a significant impact on polymyxin B clearance, and whether the dose should be decreased in patients with renal impairment. The current FDA-approved dosing recommendation for polymyxin B in the label sheet states that the dose should be decreased in patients with renal insufficiency, but no specific recommendations on the dose adjustment was provided. However, the current guidelines for polymyxin use issued in 2019 do not suggest the reduction of dose for patients with renal insufficiency (Tsuji et al., 2019). Nevertheless, the guideline acknowledges that larger PK studies are needed to validate the influence of renal function on the exposure. Previously, several PK studies have suggested the clearance of polymyxin B was not associated with CrCL (Sandri et al., 2013; Kubin et al., 2018; Manchandani et al., 2018; Miglis et al., 2018). However, recent PK studies performed in Chinese patients all identified CrCL as a significant covariate on polymyxin B clearance (Wang et al., 2020; Li et al., 2021; Yu et al., 2021). Wang et al. (2020), Yu et al. (2021) defined CrCL as a significant impactor on polymyxin B clearance in critically ill patients. Meanwhile, Li et al. (2021) verified it in renal transplant recipients, and suggested dose reduction was needed in patients with renal insufficiency. The impact of renal function on polymyxin B clearance seemed to be more significant in Chinese species. Given the conflicting findings from current studies, larger prospective studies are needed to validate the renal function involvement and ethnic variations in polymyxin B clearance.

The association between total body weight and polymyxin B clearance was also assessed in this study. Similar to other studies (Kubin et al., 2018; Manchandani et al., 2018; Wang et al., 2020; Li et al., 2021; Yu et al., 2021), we did not identify body weight as a significant covariate in polymyxin B PK profiles in lung transplant recipients. In addition, models that allowed the allometric exponent for weight adjustment did not produce better fits to the data. Notably, dosage strategy based on weight is currently recommended by the guideline (Tsuji et al., 2019). However, Miglis et al. (2018) pointed out that in the weight-based dosing strategy, patients with extremely low body weight were at risk of subtherapeutic exposure, while patients with extremely high body weight were at risk of toxicity exposure. Future studies are needed to address whether or what weight-based dosing strategies were suitable for patients with extremely low or high body weight.

Previously, we investigated the pharmacokinetics of polymyxin B in critically ill patients (Yu et al., 2021). In that study, we assessed the effect of co-inhalation of polymyxin B on the blood exposure and its pharmacokinetics, but co-inhalation therapy was not included as a covariate in the final model, which indicated the inhalation therapy with dose regimen of 25 mg q12h did not significantly increase the exposure of polymyxin B in blood. In this study, as all of the included patients suffered CRO-caused pneumonia, polymyxin B was administered intravenously and by inhalation to optimize polymyxins according to the guideline (Tsuji et al., 2019), the effect of inhalation therapy on the blood exposure therefore could not be assessed in lung transplant recipients. However, the recommended intravenous dose regimen based on the pharmacokinetic profile of polymyxin B in lung transplant recipients can be interpreted for those patients with other indications. To the best of our knowledge, the significant drug-drug interaction of polymyxins has not been reported yet. In this study, we investigated the effect of concomitant with furosemide or albumin on the PK of polymyxin B, to assess whether the change in the quantity of body fluid caused by furosemide and PPB change caused by albumin had an impact on the PK of polymyxin B. The result showed concomitant with furosemide or albumin did not significantly affect the PK of polymyxin B, but which might be because of limited number of the included patients.

Polymyxin B sulfate was reapproved for clinical use by the Chinese national medical products administration (NPMA) in 2017. Therefore, the data on its clinical efficacy and adverse events in Chinese patients is limited. In our study, we enrolled 34 CRO-caused pulmonary infections of lung transplant recipient. All patients had the treatment with polymyxin B administered intravenously and combined with inhalation of polymyxin B at the dose of 25 mg q12h. 29 (85.29%) patients were considered of clinical cure or improved. The clinical efficacy of polymyxin B in the current study was similar to the efficacy of polymyxin B in other transplant patients reported previously (Mostardeiro et al., 2013; Wen et al., 2022). However, a relatively lower nephrotoxicity rate of polymyxin B (14.71%) was observed in our study compared to those reported in renal transplant patients, since kidney transplant patients are prone to renal dysfunction (Mostardeiro et al., 2013; Wen et al., 2022). However, a recent study performed by Li et al. (2021) in renal transplant patients showed an extremely low incidence of AKI, as they used a relatively lower maintenance dose of 40 mg q12h. The nephrotoxicity of polymyxin B is a dose-dependent adverse effect (Ojo et al., 2003). In addition, 55.88% of patients in our study developed skin hyperpigmentation. Several reports suggested a possible association between long-term polymyxin B exposure and hyperpigmentation (Knueppel and Rahimian, 2007; Bergamasco et al., 2012; Zavascki et al., 2016). Most patients could have a complete recovery of the previous skin color in 3–6 months (Zavascki et al., 2016).

This is the first study to simulate the PTA and AUC_ss,24h_ of different dosage regimens in lung transplant patients. *In vitro* and animal studies pointed out that *f*AUC/MIC is the PK/PD index that is best correlated with the efficacy of polymyxins (Tsuji et al., 2019). In the thigh infection model, the *f*AUC/MIC values of colistin for 2log_10_ bacterial killings were approximately 20 for *P. aeruginosa* and *Acinetobacter Baumannii* (Cheah et al., 2015). Therefore, considering the similar molecular structures and *in vitro* activity of colistin and polymyxin B, the fAUC/MIC ≥20 was determined as the PK/PD target in our Monte Carlo simulations. Our result showed that for pathogen at MIC = 1 mg/L, the recommended polymyxin B dosage with 50 mg q12h in its label was insufficient for the patients with CrCL >50 ml/min, and a higher maintenance dose of 75 mg q12h may be the alternative to achieve the PKPD target as well as maintain the exposure within the safety target. Moreover, similar to our previous findings in critically ill patients (Evans et al., 1999), no dose recommendation was made for MIC at the current EUCAST breakpoint of 2 mg/L, because a high daily dose of 100 mg q12h increased the risk of nephrotoxicity despite it could achieve the PTA target in patients with CrCL <50 ml/min. Thus, the determination of optimal dosage strategy should be based on the balance between toxicity and the need for early efficacious exposure.

There are some limitations in the present study. Firstly, this study enrolled a relatively small number of patients, leading to a lack of external validation of the PK model. Second, the small sample size resulted in the limited analysis of risk factors for clinical failure and toxicity events. Third, the samples were taken 1, 2, and 6 h post-infusion, the subsequent model is not informed to characterize the PK across the entire dosing interval, which may be a reason why a 1-compartment model was selected but not a 2-compartment model. Finally, as the PK/PD target for simulations was derived from pre-clinical studies, the PTA endpoints of dose regimens should be further confirmed clinically.

In general, this is the first study that investigated the pharmacokinetics of polymyxin B in lung transplant recipients. Renal function significantly affects the clearance of polymyxin B, and an adjusted dosage regimen might be needed in patients with renal insufficiency. In addition, the currently recommended dose regimens by the label sheet of polymyxin B may generate a subtherapeutic exposure for MIC = 2 mg/L, whereas the high maintenance dose of l00 mg q12h would increase the risk of nephrotoxicity.

## Data Availability

The original contributions presented in the study are included in the article/Supplementary Material, further inquiries can be directed to the corresponding authors.
