# Supplementary material for: Population pharmacokinetic analysis, renal safety, and dosing optimization of polymyxin B in lung transplant recipients with pneumonia: A prospective study
Source: Front Pharmacol. 2022 Oct 13;13:1019411. doi: 10.3389/fphar.2022.1019411 (PMC9608142; doi:10.3389/fphar.2022.1019411)
Supplement: Supplementary file 1 [file DataSheet1.docx]

**Table S1 Population pharmacokinetic parameter estimates from the base model**

| **Parameter** | **Estimate** | **RSE** | **shrinkage** |
| --- | --- | --- | --- |
| **Fixed Effects** | | | |
| TVCL[L/h] | 1.65 | 9% |  |
| TVV[L] | 14.6 | 11% |  |
| **Between-subject Variability (BSV^a^)** | | | |
| BSV_CL [%CV] | 44% | 10% | 5% |
| BSV_V [%CV] | 42.2% | 16% | 28% |
| **Residual Variability (RV)** | | | |
| Proportional Error [%CV] | 38.6% | 15% | 11% |

^a^BSV calculated as $\sqrt{e^{\omega^{2}}-1}$

RSE, relative standard error; TVCL, typical value of clearance; TVV, typical value of volume.

**Table S2.** **Comparison of pharmacokinetic parameters of polymyxin B from different studies**

| **Study** | **Subject characteristics** | **Study design** | **Patients**  **number** | **Structural model** | **PK formulas** | **PK Parameters** |
| --- | --- | --- | --- | --- | --- | --- |
| **Present study** | Lung transplant patients | Prospective | 34 | 1 CMT | CL (L/h) = 1.72+(CrCL/78.49)^0.681  V (L) = 14.4 | CL (L/h): 1.87 ± 0.67  V (L): 14.4 |
| Li et al. 2021 [14] | Renal transplant patients | Prospective | 50 | 1 CMT | CL (L/h) = 1.18×（CrCL/22.2)^0.14  V (L) = 12.09 | NA |
| Sandri et al. 2013 [22] | Critically ill patients | Prospective | 24 | 2 CMT | CL (L/h/kg) = 0.0276×(TBW/75)^0.75  V1 (L/kg) = 0.0939  V2 (L/kg) = 0.330 CLic (L/h/kg) = 0.146 | NA |
| Yu et al. 2021 [26] | Adult critically ill patients | Retrospective | 32 | 1 CMT | CL (L/h) = 1.59+(CrCL/80)^0.408  V (L) = 20.5 | CL (L/h): 1.75 ± 0.43  V (L): 20.5 |
| Wang et al.  2020 [27] | Adult patients | Prospective | 46 | 2 CMT | CL (L/h) = 1.786×(CrCL/105.9)^0.362  V (L) = 6.218 V2 (L) = 11.922 Q (L/h) =13.518 | CL (L/h): 1.786 (median) |
| Miglis et al. 2018 [28] | Adult patients | Retrospective | 52 | 2 CMT | CL (L/h) = 2.63×(TBW/75)^0.75  Vc (L) = 33.77  Vp (L) = 78.20  Q (L/h) 2.32 | CL (L/h): 2.63 ± 1.41  V (L): 33.77 ± 15.21 |
| Manchandani et al. 2018 [29] | Adult patients | Prospective | 35 | 1 CMT | CL (L/h) =2.5  V (L) = 34.3 | CL (L/h): 2.5 (mean) |
| Kubin et al. 2018 [31] | Adult patients | Retrospective | 43 | 1 CMT | CL (L/h) = 2.37  V (L) = 33.4 | NA |

NA: Not available.

**Figure S1**


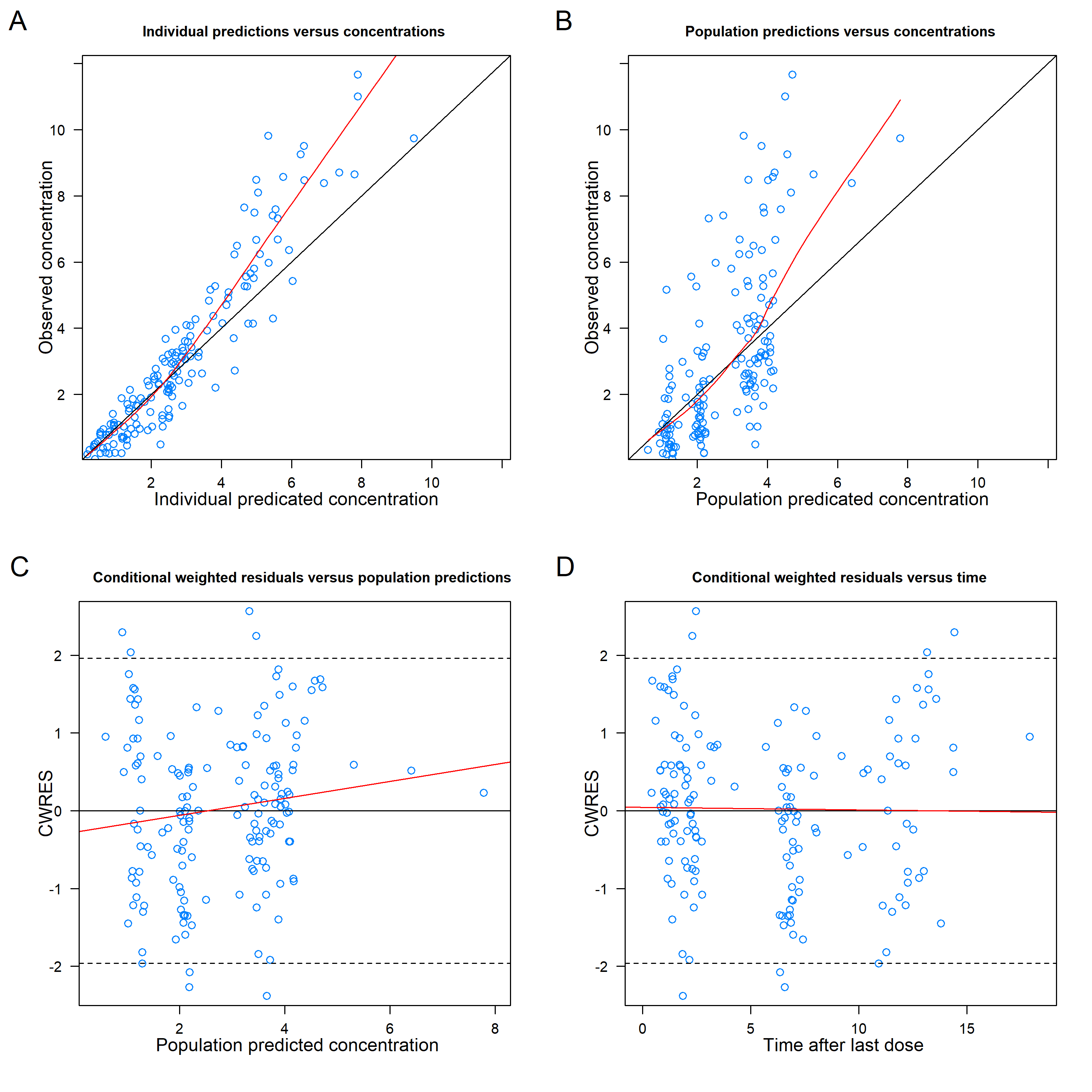


Figure S1. Goodness-of-fit plots of the base model.

(A) Observed concentration (DV) versus individual predicted concentration (IPRED); (B) DV Versus population predicted concentration (PRED); (C) conditional weighted residuals (CWRES) versus PRED; and (D) CWRES Versus time after last dose. The red solid lines in A and B are identity lines, and the red solid lines in C and D are zero lines.

**Figure S2**


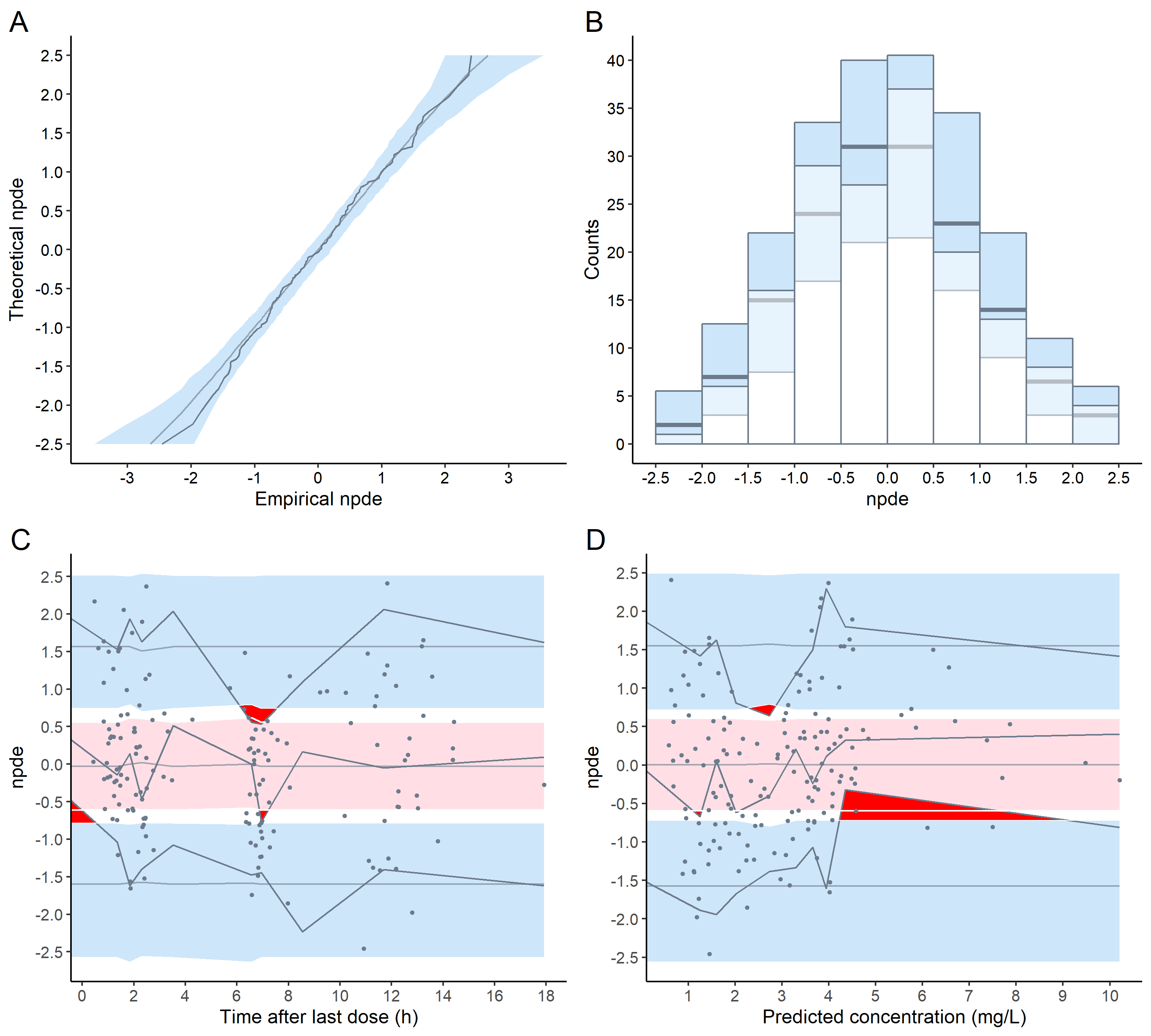


Figure S2. Normalized prediction distribution error (NPDE) plot of the final model. (A) Quantile–quantile plot of the distribution of NPDE against the theoretical distribution, (B) Histogram of the distribution of NPDE against the theoretical distribution, (C) plots of NPDE versus time after last dose; (D) plots of NPDE versus population predicted concentrations.
